# Smear layer removal comparing conventional irrigation, passive ultrasonic irrigation, EndoActivator System, and a new sonic device (Perfect Clean System) by scanning electron microscopy: An ex vivo study

**DOI:** 10.1371/journal.pone.0314940

**Published:** 2024-12-27

**Authors:** Bruna Fernanda Alionço Gonçalves, Divya Reddy, Ricardo Machado, Paulo César Soares Júunior, Sérgio Aparecido Ignácio, Douglas Augusto Fernandes Couto, Karine Santos Frasquetti, Vânia Portela Ditzel Westphalen, Everdan Carneiro, Ulisses Xavier da Silva Neto

**Affiliations:** 1 Department of Endodontics, School of Health and Biosciences, Pontifical Catholic University of Paraná –PUC/PR, Curitiba, Paraná, Brazil; 2 Advanced Standing for International Dentist Program—ASPID Program, Health Sciences Center, College of Dentistry, University of Oklahoma, Oklahoma City, Oklahoma, United States of America; 3 Health Sciences Center, Division of Endodontics, Department of Restorative Sciences, College of Dentistry, University of Oklahoma, Oklahoma City, Oklahoma, United States of America; 4 Department of Materials Science, Polytechnical School, Pontifical Catholic University of Paraná –PUC/PR, Curitiba, Paraná, Brazil; 5 Department of Biostatistics, School of Health and Biosciences, Pontifical Catholic University of Paraná –PUC/PR, Curitiba, Paraná, Brazil; University of Puthisastra, CAMBODIA

## Abstract

**Aim:**

This study evaluated the smear layer removal provided by conventional, sonic, and ultrasonic irrigation techniques.

**Methodology:**

Forty extracted human mandibular first premolars were selected and instrumented using the ProTaper Next System files and 2.5% sodium hypochlorite. Afterward, they were divided into 4 groups (n. 10) according to the irrigation technique used to perform the final irrigation with a chelating solution (17% EDTA): conventional irrigation (CI), passive ultrasonic irrigation (PUI), EndoActivator System (EAS), and Perfect Clean System (PCS). The smear layer removal was determined through a score after evaluating scanning electron microscope images (1.000x) obtained at 1, 5, 8, and 12mm from the working length (WL). Statistical analyses were carried out by the Kruskal-Wallis and Dunn’s tests with a significance level of 5% (P < 0.05).

**Results:**

All irrigation techniques were unable to promote an effective smear layer removal at 1mm from the WL in comparison with the other locations (P < .05). At 5, 8, and 12mm from the WL, no statistically significant differences were observed among CI, PUI, EAS, and PCS (P > 0.05). At 12mm from the WL, statistically significant differences were only identified after comparing PCS and CI (P < .05).

**Conclusion:**

The smear layer removal was only efficient at 5, 8, and 12 from the WL with no significant statistical differences among CI, PUI, EAS, and PCS (P > 0.05).

## Introduction

Chemomechanical preparation is responsible for shaping and cleaning the root canal system (RCS) using endodontic files and auxiliary chemical substances [1]. The technical and scientific evolution experienced by Endodontics in the last few years has allowed the development of instruments, techniques, and devices that enable the biomechanical preparation of root canals to be carried out faster and more comfortably for both patient and clinician. However, the anatomical complexity of the RCS keeps favoring the existence of areas not touched by the endodontic files [2], compromising the main biological objectives of the chemomechanical preparation–the cleaning and disinfection processes. Another important issue is that the associated use of endodontic instruments and irrigating solutions invariably promotes the production of "a material" composed of dentin, odontoblastic processes, pulp tissue, and bacteria called smear layer [3]. The smear layer might induce persistent infection due to the presence of organic and inorganic substrates in its composition, hindering the performance of the irrigating solutions [4] and blocking the entrances of dentinal tubules, preventing the penetration of intracanal medications [5] and endodontic sealers, mainly in the final millimeters of the RCS [3, 6].

Agitation of sodium hypochlorite (NaOCl) and its continuous renewal affords an uninterrupted source of nascent chlorine, optimizing tissue dissolution [7–9]. Bearing this in mind, sonic, ultrasonic, and negative pressure devices have been developed and studied to improve the chemical cleaning and smear layer removal from the RCS [10–14].

Despite favorable results reached by passive ultrasonic irrigation (PUI), important drawbacks are associated with the technique. When an ultrasonic tip touches the root canal walls, it dampens the energy. It constrains the file movement, and file-to-wall contact occurs approximately 20% of the time [15]. As these tips are generally made of metal alloy, their contact with the root canal walls might cause uncontrolled and unnecessary dentin removal, weakening the root structure [16] and enabling the formation of "a new smear layer" [17]. Hence, other alternatives have been developed and studied [11, 13, 14].

The EndoActivator System (EAS) (Dentsply Tulsa Dental Specialties, Tulsa, OK, United States of America) is a sonically driven irrigant activation tool developed to produce vigorous fluid agitation within the RCS. It has improved irrigation efficacy compared to conventional irrigation (CI). EAS comprises a portable handpiece and three disposable flexible polymer tips of distinct sizes (15/.02, 25/.04, and 35/.04), which do not cause wear to the intracanal dentin [10, 11].

Perfect Clean System (PCS) (Microdont, São Paulo, SP, Brazil) is a new sonic device recently developed and launched. According to the manufacturer, it works from an electromechanical set promoting intense vibrations responsible for the agitation of chemical solutions into the RCS, favoring better cleaning and disinfection processes and higher penetration of intracanal medications and endodontic sealers into the dentinal tubules. PCS presents three flexible polymer tips: fine (15/.02), medium (25/.04), and coarse (35.04) [18]. Currently, there is no research regarding the effectiveness of PCS in removing the smear layer.

To the best of our knowledge, this is the first study planned to evaluate the removal of the smear layer in 4 different root canal levels (1, 5, 8, and 12mm from the working length [W]), comparing CI, PUI, EAS, and PCS by scanning electron microscopy images (SEM). The null hypothesis established was that there is no difference in the smear layer removal considering the root canal levels and the irrigation systems investigated.

## Materials and methods

**Fig 1:**
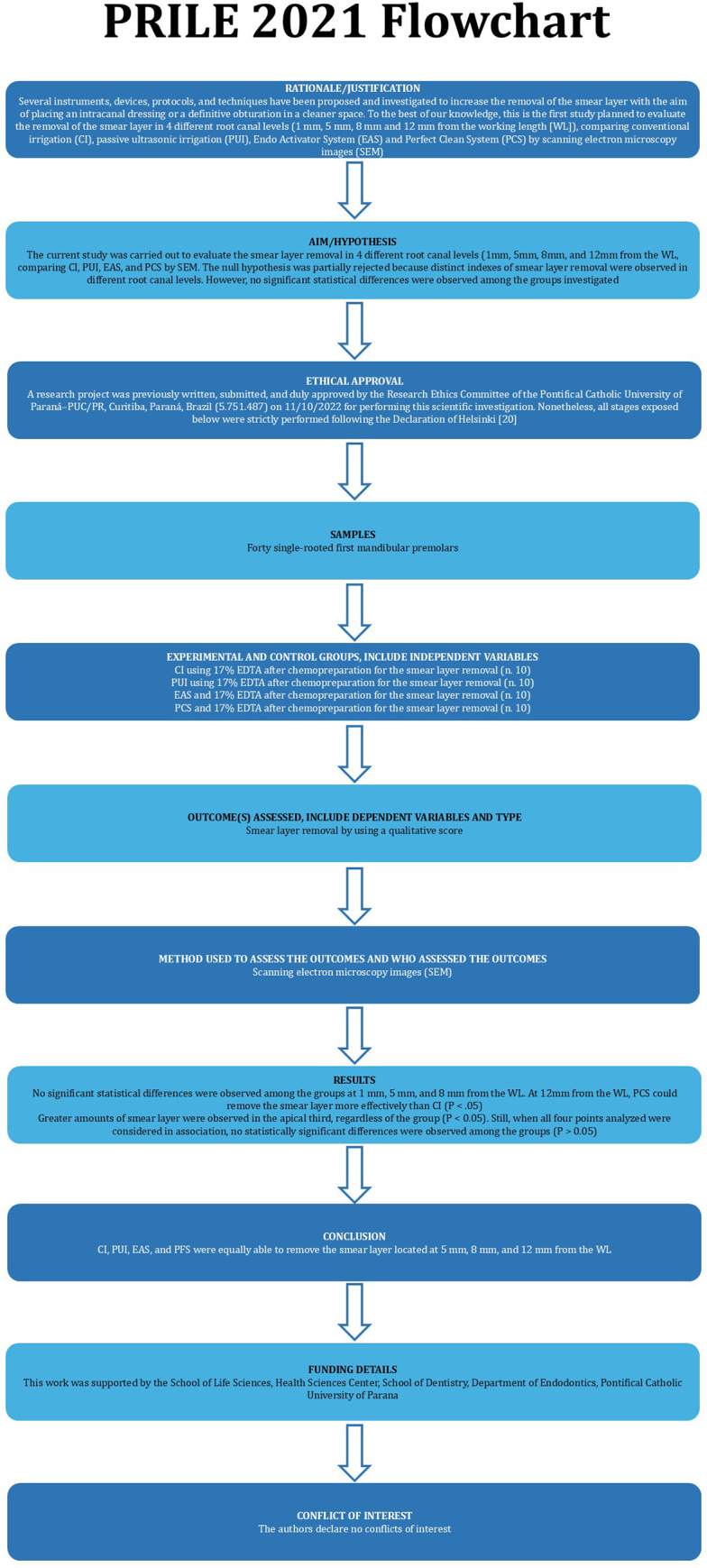
PRILE 2021 flowchart.

This manuscript was written following PRILE guidelines (Fig 1) [19].

### Approval by the research ethics committee

A research project was previously written, submitted, and duly approved by the Research Ethics Committee of the Pontifical Catholic University of Paraná –PUC/PR, Curitiba, Paraná, Brazil (5.751.487), on 11/10/2022 for performing this scientific investigation. Nonetheless, all stages exposed below were strictly performed following the Declaration of Helsinki [20].

### Sample size calculation

The sample size calculation was performed using the g G*Power version 3.1 software for Mac (Heinrich Heine, Universität Düsseldorf, Düsseldorf, Germany) by selecting the analysis of variance (ANOVA) test using data from a specific previously performed pilot study. The effect size for the present research was 1.66, the alpha type error was 0.05, the beta power was 0.80, and the radius was 1. Hence, a total of 9 specimens per group were needed. To account for specimen loss, the number of specimens was increased by 10% per group. Thus, ten teeth were used per group [21, 22].

### Specimens’ selection

Ninety single-rooted first mandibular premolars provided by the Teeth Bank of the Pontifical Catholic University of Paraná –PUC/PR, Curitiba, Paraná, Brazil, were initially selected from 11/11/2022 to 11/18/2022. All of them were cleaned and digitally radiographed in the mesiodistal and buccolingual directions to prove the presence of a single canal and the absence of previously performed root canal treatment, root resorptions, and anatomical complexities (Vistascan Mini, Dürr Dental, Bietigheim-Bissingen, Germany) according to the following parameters: 70 kV, 8 mA, and exposition time of 0.32s [23]. Mesiodistal radiographs were also used to measure the distances between the buccal and lingual root canal walls at 1mm, 5mm, 8mm, and 12mm from the working length (WL). Sixty specimens presenting the most similar anatomical features were submitted to cone-beam computed tomography (CBCT) scans to establish the root canals’ volume, surface area, and structure model index. All CBCT scans were obtained using the PreXion 3D device (Model XP 68, Yoshida Dental Mfg Co, Ltd, Tokio, Japan). The acquisition parameters were as follows: a 0.14-mm voxel size, 90 kV, 4 mA, and 19 seconds (normal mode) with a field of view limited to 80 x 80 mm. The images were analyzed using the PreXion 3D Viewer software (Prexion, Inc, San Mateo, CA, United States of America)) on a Dell Precision T5400 workstation with a 17inch Dell LCD screen with a resolution of 1280 x 1024 pixels with 85 Hz and 0.255-mm dot pitch (Dell, Round Rock, TX, United States of America) operated at 24 bits in a dark environment. Contrast, brightness, sharpness, and zoom were adjusted using the software’s tools to ensure better viewing. The choice of the 40 specimens included in the sample was performed considering the following parameters: i) volume average/inferior limit–superior limit (mm^3^): 11.18/3.61–32.44. ii) surface area average/inferior limit–superior limit (mm^2^): 50.77/10.79–98.04. iii) structure model index average/inferior limit–superior limit: 2.52/0.21–3.90 [24]. Later, all specimens were kept in 0.1% thymol solution until use (Fórmula & Ação, São Paulo, SP, Brazil).

### Specimens’ preparation

The crowns were initially sectioned with a low-speed steel cutting disc (Isomet-Buehler, Lake Bluff, IL, United States of America), obtaining roots 15mm in length [25]. To prevent the apical extrusion of the irrigating solution, the apex of the specimens was covered with OpalDam (Ultradent Products, South Jordan, UT, United States of America) [26]. During this procedure, a # 15 K-Flexofile (Dentsply-Maillefer, Ballaigues, Switzerland) was inserted into the root canal up to the apical foramen to avoid the penetration of the material. The root canal entrance was prepared using 3082 (KG Sorensen, Barueri, SP, Brazil) and n. II Largo drills (Dentsply-Maillefer). Cervical and middle root canal thirds were prepared using an Orifice Shaper file 17/.08 (MK Life, Porto Alegre, RS, Brazil). After, the WL was established by subtracting 1mm from the point where a # 15 K-file (Dentsply-Maillefer) was visible at the apical foramen. The anatomical diameter was determined by inserting #10, #15, #20, and #25 K-files (Dentsply-Maillefer) in ascending order until the first one fits the root canal at the WL [27]. Only specimens presenting anatomical diameters corresponding to a # 20 K file were kept in the sample. Specimens presenting different anatomical diameters were replaced.

Chemomechanical preparation was carried out using the ProTaper Next files (Dentsply Sirona, Charlotte, NC, United States of America) powered by an E-Connect electric motor (MK Life) according to the manufacturer’s instructions until the X4 file. Irrigation was conducted at each file change or use, using 2.5 ml of 2.5% NaOCl (Fórmula & Ação) and a NaviTip 31G double side-port needle (Ultradent, Indaiatuba, SP, Brazil), calibrated at 1mm from the WL, matched to a plastic syringe. The apical patency was maintained with a # 15 K-file (Dentsply‑Maillefer). A total of 20 ml of irrigating solution was used during the chemomechanical preparation [14]. Afterward, the specimens were randomly divided into four groups (n. 10) according to the different techniques for performing the final irrigation using a chelating solution (17% EDTA, Formula & Ação) (Table 1).

**Table 1:** Group distribution according to the activation methods investigated.

Groups	Activation protocol
**CI** *	CI was performed using a 31‑gauge double side-port needle (Ultradent) calibrated at 1mm from the WL matched to a plastic syringe. Nine cycles of 20s were carried out, applying small vertical movements of around 3 mm, totaling an agitation and action time of 3 min. The root canal was rinsed with 2.5 mL of 17% EDTA (Fórmula & Ação) during each cycle.
**PUI#** **	PUI was performed using a 20/01 ultrasonic irrigation file (Irrisonic E1, Helse, Santa Rosa de Viterbo, SP, Brazil), calibrated at 1mm from the WL, adjusted on 15% of power (Endodontics mode) powered by an ultrasonic device (Jet Sonic, Gnatus, São Paulo, SP, Brazil) by nine cycles of 20s totaling an agitation and action time of 3min. Before each cycle, the root canal was rinsed with 2.5 mL of 17% EDTA (Fórmula & Ação).
**EAS#** ***	Sonic activation was carried out using the large polymer tip (35.04) of EAS (Dentsply Tulsa Dental Specialties), calibrated at 1mm from the WL, by nine cycles of 20s, totaling an agitation and activation time of 3min. Before each cycle, the root canal was rinsed with 2.5 mL of 17% EDTA (Fórmula & Ação).
**PCS#** ****	Sonic activation was conducted using the large polymer tip (35.04) of PCS (Microdont), calibrated at 1mm from the WL, by mine cycles of the 20s, totaling an agitation and action time of 3 min. Before each cycle, the root canal was rinsed with 2.5 mL of 17% EDTA (Fórmula & Ação).

#The root canal was initially filled using 2.5mL of 17% EDTA using a 31‑gauge double side-port needle (Ultradent) calibrated at 1mm from the WL matched to a plastic syringe.

*Conventional irrigation.

**Passive ultrasonic irrigation.

***EndoActivator System.

****Perfect Clean System.

After the final irrigation, the root canal was irrigated with 2.5 mL of 2.5% NaOCl (Fórmula & Ação) and dried with ProTaper Next X4 absorbent paper points (Dentsply Sirona).

### SEM analyses

**Fig 2:**
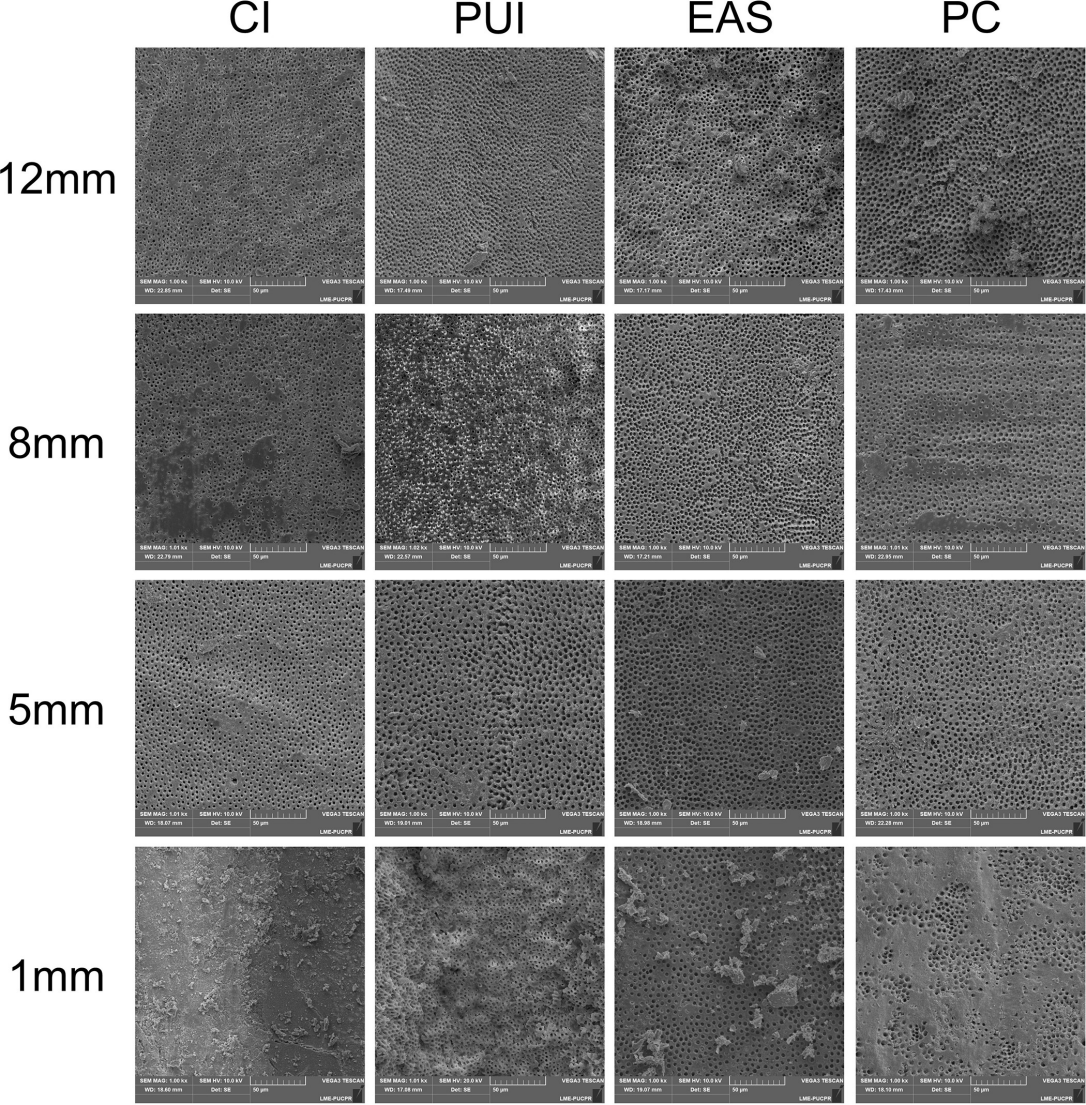
Representative SEM images of groups and places investigated.

Initially, an X4 gutta-percha cone (Dentsply Sirona) was introduced into the canal, and vertical grooves were made on the mesial and distal external surfaces of each root with double-sided diamond discs (KG Sorensen), operated at low rotary speed until the gutta-percha cone could be seen. This procedure facilitated the root’s fracture into two halves [25, 27]. Special attention was needed during this stage to avoid accidental contamination and the movement of sharp debris into the root canal [26, 28]. The hemisections presenting better visualization were fixed on circular metal stubs to sputter coat the surface with a 30-nm layer of gold (Balzers SCD030, Oerlikon Balzers, Balzers, Liechtenstein, Germany). For each hemissection, images with 1000x magnification were obtained at 1, 5, 8, and 12mm from the WL using a scanning electronic microscope (Tescan VEGA 3, Tescan, Brno, Czech Republic), totaling 160 images (Fig 2). No specimens were lost during the investigation.

Three examiners not involved with the methodological design of the study were previously calibrated by analyzing 32 images randomly selected from two specimens of each group, using the scoring system proposed by Gambarini and Laszkiewicz (Table 2) [29]: **Score 1:** no smear layer, dentinal tubules open; **Score 2:** small amount of smear layer, some dentinal tubules open; **Score 3:** homogenous smear layer covering the root canal wall, only a few dentinal tubules open; **Score 4:** complete root canal wall covered by a homogenous smear layer, no open dentinal tubules; **Score 5:** heavy, non-homogenous smear layer covering the complete root canal wall. During the calibration process, examiners’ communication was allowed to determine a single score for each image. The definitive analysis was performed without communication among the examiners [6].

**Table 2:** Gambarini and Laszkiewicz [29] proposed a scoring system to classify the removal of smear layers.

Score	Qualitative description
1	There is no smear layer and open dentinal tubules.
2	A small amount of smear layer, with some open dentinal tubules.
3	A homogenous smear layer covers the root canal wall, with only a few open dentinal tubules.
4	A homogenous smear layer with no open dentinal tubules covers the root canal wall.
5	Heavy, non-homogenous smear layer covering the complete root canal wall.

### Statistical analysis

The smear layer removal scores provided by the examiners were tabulated in a spreadsheet using the Excel software (Microsoft Corp., Redmond, WA, United States of America). The agreement level among examiners was assessed by the Kappa test, which presented an overall result of 0.90. The non-parametric Kruskal-Wallis test for independent samples was used to compare the scores for the groups and root canal levels and the occurrence of a potential interaction among them. After identifying significant statistical differences between the variables analyzed, multiple pairwise comparisons were carried out through the non-parametric Dunn’s test. Statistical analyses were conducted using SPSS 25 software (IBM, Armonk, NY, United States of America), with a significance level of 5% (P < .05) [30].

## Results

Table 3 exposes the smear layer removal provided by CI, PUI, EAS, and PCS at the four different root canal levels. No significant statistical differences were observed among the groups at 1, 5, and 8mm from the WL. At 12mm from the WL, PCS could remove the smear layer more effectively than CI (P < .05).

**Table 3:** Statistical data regarding the smear layer removal comparing the four methods evaluated at each root canal level.

Groups	1mm from the WL		5mm from the WL		8mm from the WL		12mm from the WL		P value^+^
	Mean	SD	Mean	SD	Mean	SD	Mean	SD	
**CI** ^*^	2.70^A,a^	1.33	2.20^A,a^	1.22	1.90^A,a^	0.99	2.50^A,a^	1.26	P > .05
**PUI** ^**^	2.90^A,a^	1.10	1.40^B,a^	0.51	2.00^B,a^	1.05	1.60^B,ab^	0.69	P < .05
**EAS** ^***^	2.30^A,a^	1.16	1.50^AB,a^	0.85	1.30^B,a^	0.67	1.70^AB,ab^	0.67	P < .05
**PCS** ^****^	3.30^A,a^	0.94	1.60^B,a^	1.07	1.80^B,a^	0.78	1.50^B,bc^	0.97	P < .05
**P value** ^**+**^	P > .05		P > .05		P > .05		P < 0.05		

#Capital letters correspond to rows.

##Small letters correspond to columns.

*Conventional irrigation.

**Passive ultrasonic irrigation.

***EndoActivator System.

****Perfect Clean System.

^+^Determined by the non-parametric Dunn’s test (P < .05).

Table 4 exposes the smear layer removal provided by each system/group in the four distinct points in the root canals. In the specimens submitted to CI, smear layer removal was similar in the different root canal thirds. When PUI was used, statistically significant differences were only observed between 1 and 5mm (P = 0.00) and 1 and 12mm (P = 0.01) from the WL. Statistical differences were only significant between 1 and 8mm (P = 0.02) from the WL after using EAS. In the specimens submitted to PCS, all evaluated sites presented greater smear layer removal compared to the final millimeter of the root canal: 1 and 5mm from the WL (P = 0.00); 1 and 8mm from the WL (P = 0.00), and 1 and 12mm from the WL (P = 0.00).

**Table 4:** A pairwise comparison considers the root canal levels at each root canal level.

**CI** *	**Pairwise comparison considering root canal levels**		**P value** ^**+**^
	1mm from the WL	5mm from the WL	0.38
	1mm from the WL	8mm from the WL	0.18
	1mm from the WL	12mm from the WL	0.77
	5mm from the WL	8mm from the WL	0.65
	5mm from the WL	12mm from the WL	0.56
	8mm from the WL	12mm from the WL	0.30
**PUI** **	**1mm from the WL**	**5mm from the WL**	**0.00**
	1mm from the WL	8mm from the WL	0.08
	**1mm from the WL**	**12mm from the WL**	**0.01**
	5mm from the WL	8mm from the WL	0.23
	5mm from the WL	12mm from the WL	0.64
	8mm from the WL	12mm from the WL	0.45
**EAS** ***	1mm from the WL	5mm from the WL	0.08
	**1mm from the WL**	**8mm from the WL**	**0.02**
	1mm from the WL	12mm from the WL	0.30
	5mm from the WL	8mm from the WL	0.64
	5mm from the WL	12mm from the WL	0.48
	8mm from the WL	12mm from the WL	0.24
**PCS** ****	**1mm from the WL**	**5mm from the WL**	**0.00**
	**1mm from the WL**	**8mm from the WL**	**0.00**
	**1mm from the WL**	**12mm from the WL**	**0.00**
	5mm from the WL	8mm from the WL	0.45
	5mm from the WL	12mm from the WL	0.86
	8mm from the WL	12mm from the WL	0.36

*Conventional irrigation.

**Passive ultrasonic irrigation.

***EndoActivator System.

****Perfect Clean System.

^+^Determined by the non-parametric Dunn’s test (P < .05).

Greater amounts of smear layer were observed in the apical third, regardless of the group (P < 0.05). Still, when all four points analyzed were considered in association, no statistically significant differences were observed among the groups [P > 0.05] (Fig 2).

## Discussion

Despite the lack of clinical evidence about the smear layer’s impacts on endodontic prognosis, its removal has been recommended so that cleaner root canals may be accurately filled with intracanal medication or definitively obturated [3, 31]. Thus, several instruments, devices, protocols, and techniques have been proposed and investigated to increase the removal of the smear layer [12, 32–34]. The current study was carried out to evaluate the smear layer removal in 4 different root canal levels (1mm, 5mm, 8mm, and 12mm from the WL) comparing CI, PUI, EAS, and PCS by SEM. The null hypothesis was partially rejected because distinct indexes of smear layer removal were observed in different root canal levels. However, no significant statistical differences were observed among the groups investigated.

### Study methodology and results

Strict criteria were applied during the selection of the specimens for this investigation. Mandibular first premolars were chosen because they generally present relatively simple anatomy (single root canals and single main apical foramina in 75.8% and 78.9%, respectively) [35]. First, 90 teeth provided by the Teeth Bank of the Pontifical Catholic University of Paraná –PUC/PR, Curitiba, Paraná, Brazil were selected. All specimens were cleaned and radiographed in the mesiodistal and buccolingual directions to prove the presence of a single canal and the absence of previously performed root canal treatment, root resorptions, and anatomical complexities. The same radiographs measured the distances between the root canal walls at 1mm, 5mm, 8mm, and 12mm from the WL. Sixty specimens presenting the most similar anatomical features were submitted to CBCT scans, resulting in the selection of 40 specimens considering the following parameters: i) volume average/inferior limit–superior limit (mm^3^): 11.18/3.61–32.44. ii) surface area average/inferior limit–superior limit (mm^2^): 50.77/10.79–98.04. iii) structure model index average/inferior limit–superior limit: 2.52/0.21–3.90 [24]. Still, after flaring the cervical and middle root canal thirds, the anatomical diameters were determined [27], and only specimens with anatomical diameters corresponding to a n. 20 K-files were kept in the sample. These efforts were carried out to limit the impacts of the root canal anatomy and dentinal morphology. The use of specimens presenting similar anatomical profiles and root canal dimensions likely allowed the performance of more reliable analysis, considering that after the root canal instrumentation, the areas investigated presented comparable amounts of dentinal tubules with resemblant diameters, considering both dentinal morphological features (quantity and diameters of dentinal tubules) decrease near the external root surface. A sealed apex model was also employed to replicate a clinical situation aiming at the occurrence of the vapor-lock phenomenon [11].

SEM is the most employed technique for assessing smear layer removal [36, 37]. However, this approach has faced criticism due to the limited areas examined compared to the full extent of the root canal. In addition, assigning scores to classify cleanliness levels might be considered a subjective measure, dependent on the varying interpretations of individual examiners [38, 39]. To mitigate the subjectivity effects, in the present research, images were obtained at lower magnifications (×1,000) [11] compared to previous studies– ×2,000 [40] and ×1,500 [41]–allowing for a more thorough evaluation of the four regions investigated in each specimen. Furthermore, a thorough calibration process was conducted beforehand [6, 11], resulting in Kappa values of 0.90 or higher, indicating strong inter-examiner agreement and underscoring the significance of calibration in ensuring the accuracy and consistency of the results obtained [6].

Formerly conducted research normally used three root canal locations to obtain representative images from the three root canal thirds [27, 42–44]. In the current investigation, images at 1mm from the WL were also included due to the proximity to the apical foramen, which is a challenging area for achieving an ideal and accurate cleaning and disinfection process [45, 46]. At this level, the smear layer removal was extensively compromised compared with the other locations (P < .00) (5, 8, and 12mm from the WL), which did not statistically differ among them (P > .05). Regarding CI, this outcome might be attributed to the pronounced constriction in the apical third, which hinders the movement and circulation of the irrigants and chelating agents, thus affecting smear layer removal [6]. Regarding the other groups, it is important to note that activation techniques improve the cleaning by directing the solution against the dentinal walls. However, the contact of the activating instruments with the root canal walls may lead to the formation of "a new smear layer," mainly in the apical root canal third. Kanaan et al. [17] evaluated this hypothesis using PUI, Easy Clean (BassiEndo, Belo Horizonte, BH, Brazil), and EDDY (VDW, Munich, Germany). The specimens (mandibular premolars) and finishing file tips were the same as used herein (R40). The formation of “a new smear layer” was proved after using the three activation systems, even in specimens not submitted to the root canal instrumentation.

Another important finding from the present research is that the PCS provided statistically significantly better results than CI at 12mm from the WL. This result might be relevant regarding the restoration of endodontically treated teeth. Considering fiber posts have been widely used for the rehabilitation of teeth previously submitted to endodontic interventions, the effective removal of the smear layer provided by PCS might favor the dentinal tubule penetration of post-cementing agents, thus enhancing the bond strength between the fiber post and dentin in the cervical root canal third and the longevity of teeth [47]. Future studies should be conducted to evaluate these hypotheses.

The main finding of this study is the lack of significant statistical differences regarding the smear layer removal comparing PUI, sonic activation (EAS and PCS), and CI. Different results about this matter are considerably frequent. While some studies showed advantages provided by the activation techniques, others demonstrated the lack of statistically significant differences comparing activation techniques with CI or better results obtained by CI. Significant methodological differences are the main ones responsible for distinct or contrary results observed by research to analyze the same dependent variable.

As this was the first study addressing the smear layer removal provided by the PCS, other scientific investigations are needed to obtain more reliable information about its performance.

### Limitations and future perspectives

SEM is a global research method frequently employed to assess the removal of the smear layer. However, it has several limitations. Often, samples need to be sectioned for qualitative evaluation using scoring systems, which are considered a significant source of bias. In addition, although SEM can produce high-resolution images with topographical, morphological, and compositional details, it provides two-dimensional imaging and is more likely to produce artifacts [48]. Additionally, there is a potential for radiation exposure that may penetrate beneath the sample’s surface [48].

Another notable limitation of the present study is that smear layer removal was evaluated on only one root canal wall. While this focus offers valuable insights into a particular aspect, it does not diminish the importance of comprehending the conditions on that wall. Machado et al. [49] studied the residual smear layer after root canal instrumentation by using Niti, M-Wire and CM-Wire by SEM in the buccal/lingual surface and mesial/distal surfaces. Future similar studies by SEM should consider this approach.

Another point worth emphasizing in the current research is that only single-rooted first mandibular premolars with straight root canals were treated. Further studies are necessary to investigate the effectiveness of various active irrigation techniques in cleaning curved root canals [33, 42].

Based on the above, the findings of the present study should be interpreted cautiously. In vivo studies are required to further assess the impact of different irrigation systems on root canal cleaning efficiency and their influence on endodontic prognosis [50].

## Conclusions

Despite this study’s limitations, CI, PUI, EAS, and PCS were equally able to remove the smear layer located at 5 mm, 8 mm, and 12 mm from the WL.
